# The Incidence-Based Mortality and Survival Trends in Patients with Gastric Signet Ring Cell Carcinoma

**DOI:** 10.1155/2022/3308801

**Published:** 2022-05-28

**Authors:** Xiangpan Li, Yuxin Chu, Qibin Song, Qinyong Hu

**Affiliations:** Cancer Center, Renmin Hospital of Wuhan University, Wuhan 430060, Hubei, China

## Abstract

**Materials and Methods:**

The patients from the Surveillance, Epidemiology, and End Results (SEER) database were recruited to explore the incidence-based mortality and survival trends from 2000 to 2017. We further analyzed the differences in mortality and survival trends in these patients by sex and stage. We also used joinpoint software to evaluate the trends in annual percentage change (APC) for statistical significance.

**Results:**

14916 patients were collected, including 7801 (52.3%) male and 7115 (47.7%) female. We identified a single joinpoint at 2002. The overall incidence-based mortality of gastric SRC declined in America after 2002 (APC = −1.21, *P* < 0.05). In stratified analysis by sex and stage, the incidence-based mortality rate was higher in males than females. After 2002, the mortality rate decreased significantly in male (APC = −1.68, *P* < 0.05) and M0-stage patients (APC = −1.75, *P* < 0.05). In survival trend analysis, the 2-year relative survival improved in M0-stage gastric SRC, especially for males (APC = 1.14, *P* < 0.05). As for M1-stage patients, the 2-year relative survival significantly elevated in both male (APC = 3.87, *P* < 0.05) and female (APC = 5.63, *P* < 0.05) patients.

**Conclusions:**

The incidence-based mortality of gastric SRC has declined, and survival has improved in America over time. These optimistic trends may be attributed to cancer screening implementation and advances in novel treatments in the past decades.

## 1. Introduction

Gastric signet ring cell carcinoma (SRC) is a unique histological subtype of stomach adenocarcinoma. Based on the WHO histological classification, SRC is a distinct adenocarcinoma with >50% scattered cancer cells including intracytoplasmic mucin [1]. The typical morphology of SRC contains plentiful mucin in the cytoplasm, pushing the nucleus to the periphery [2]. Clinically, gastric SRC often inflicts the younger females, who are more susceptible to having distant metastasis, leading to poor survival outcomes [3]. Gastric SRC usually has more aggressive biological behavior than other subtypes of adenocarcinoma [4]. The overall prevalence of gastric cancer has gradually decreased in recent years, but the incidence of gastric SRC is still rising [5], accounting for 35%–45% of gastric adenocarcinoma cases recently [6].

Although the incidence trends of gastric SRC have been reported in recent years [7], little is known about its mortality trends. Evaluation of the mortality trends in gastric SRC is crucial because changes in cancer screening and lifestyle may well affect the death rate from cancer [8].

Moreover, the advent of novel therapies in recent years has improved the survival of many cancer patients [9]. It is still uncertain whether sex and tumor stage have influenced the survival trends of this disease over years. It is important to delineate the changing mortality and survival trends in gastric SRC so that doctors can design more tailored treatments for such patients.

The evidence of multiple risk factors for developing gastric SRC indicates that the epidemiology and clinical features of this disease may have changed in recent years. To better understand the changing patterns, we utilized the Surveillance, Epidemiology, and End Results (SEER) database to explore the changes in incidence-based mortality and survival trends in patients with gastric SRC during the span from 2000 to 2017. We focus on some clinical factors, such as sex and stage. The findings from this study may provide a comprehensive reflection on the changing epidemiology of gastric SRC over time.

## 2. Materials and Methods

### 2.1. Data Collection

We have utilized the SEER 18-registry database (2000–2017) to extract the incidence-based mortality and survival data, which was submitted in November 2019. The SEER 18 registries collect new cancer cases from 18 geographic regions [10]. These regions together cover about 28% of the US population. We have selected the primary site on the stomach. The ICD-O-3 histology code 8490/3 is recorded as signet ring cell carcinoma (SRC). The patients have either one primary only or the first of more primaries. The SEER database has its own individual summary/historic staging system to depict the tumor extension, which includes in situ, localized, regional, distant, and unstaged. In this study, localized and regional cancers were designated as M0-stage gastric SRC, while distant metastatic cancer represents M1-stage gastric SRC. Given that cancer is a publicly reportable disease in America, all the information extracted from the SEER database is exempt from ethical review.

### 2.2. Data Analysis

We adopted the R software to analyze our data. We focus on the following items: (I) the incidence-based mortality of gastric SRC in each year; (II) the incidence-based mortality changes over time for each sex of gastric SRC patients; (III) the incidence-based mortality changes over time for respective stage of gastric SRC patients; and (IV) the 2-year survival rate changes over time for each stage of gastric SRC. The remarkable changes and trends in the above items are assessed by joinpoint regression analyses. The joinpoint software employs a statistical algorithm to fit the optimal regression line reflecting the incidence trend over time. It will also intellectually recognize the best inflection points where rates change significantly. The optimal number of joinpoints is set aside for ultimate models. The annual percentage change (APC) can be figured out for each spline. We have used age-adjusted rates to reflect the joinpoint trends for statistical significance.

## 3. Results

### 3.1. Demographic Characteristics

A total of 14916 patients with gastric SRC were selected from the SEER database (2000–2017), including 7801 (52.3%) males and 7115 (47.7%) females. Additionally, 8066 (54.08%) patients were M0-stage, while 6850 (45.92%) patients were M1-stage. The clinical characteristics of those patients are displayed in Table 1.

### 3.2. Incidence-Based Mortality for Entire Cohort

**Figure 1:**
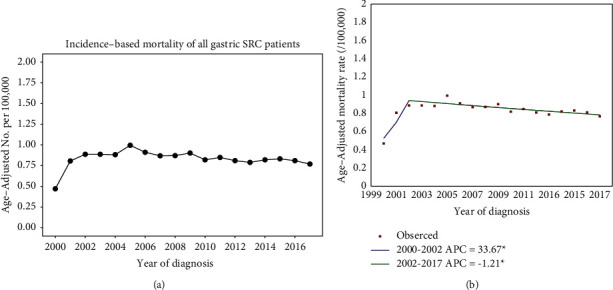
(a) The incidence-based mortality of gastric SRC over years. (b) Joinpoint analysis for the incidence-based mortality of gastric SRC in 2000–2017.

The incidence-based mortality of gastric SRC initially increased from 2000 to 2002 but subsequently remained stable or decreased (Figure 1(a)). These changes are consistent with the results of the joinpoint regression analysis. The incidence-based mortality of gastric SRC rose until 2002, with an APC = 33.67 (*P* < 0.05) (Figure 1(b)). Then the mortality rate gradually decreased, with an APC = −1.21 (*P* < 0.05). The declining trend has statistical significance, indicating that the mortality of gastric SRC has indeed abated since 2002.

### 3.3. Incidence-Based Mortality by Sex

**Figure 2:**
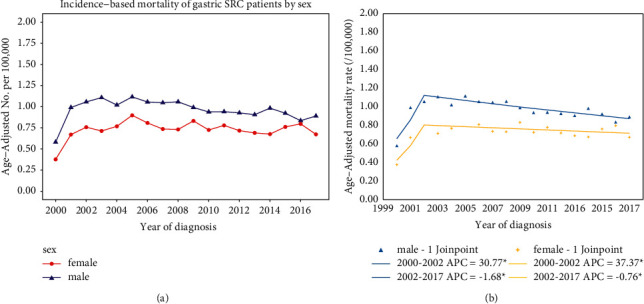
(a) The incidence-based mortality of gastric SRC by sex over time. (b) Joinpoint analysis of the incidence-based mortality of gastric SRC by sex over time.

We stratified the gastric SRC patients by sex, analyzing their incidence-based mortality trend over time. Overall, the age-adjusted mortality rate was significantly higher in males than females during the study period. As for males, the mortality rate increased from 2000 to 2003 and then gradually declined in the subsequent years. As for females, the rate likewise rose from 2000 to 2002, reached a peak in 2005 and then gradually declined (Figure 2(a)). The joinpoint regression analysis also revealed a significant sex difference in incidence-based mortality changes. In the joinpoint analysis of males, the incidence-based mortality rose by 30.77% annually from 2000 to 2002 and then steadily declined from 2002 to 2017, with an APC = −1.68 (*P* < 0.05) (Figure 2(b)). In the joinpoint analysis of females, the incidence-based mortality significantly increased by 37.37% annually from 2000 to 2002 and then gradually decreased since 2002, with an APC = −0.67 (*P* > 0.05) (Figure 2(b)). Generally, joinpoint analysis has confirmed the significant downward trend of incidence-based mortality in male gastric SRC patients after 2002.

### 3.4. Incidence-Based Mortality by Stage

**Figure 3:**
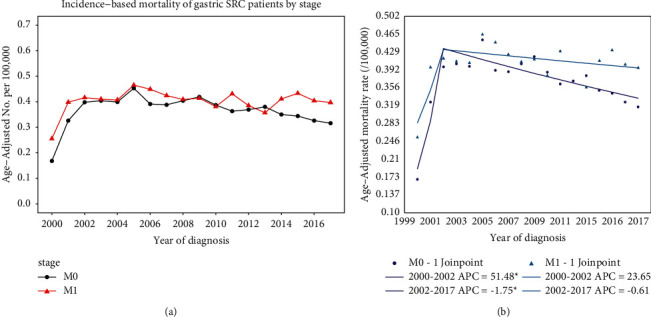
(a) The incidence-based mortality of gastric SRC by stage over time. (b) Joinpoint analysis of the incidence-based mortality of gastric SRC by stage over years.

With respect to stage, the overall trend was rising sharply until 2002. Then the M0-stage patients had a gradually decreasing trend after 2002. Comparatively, the M1-stage patients showed a relatively stable trend since 2002 (Figure 3(a)). The joinpoint analysis revealed a common inflection point at 2002 for each stage of patients. After 2002, the incidence-based mortality of M0-stage patients significantly decreased (APC = −1.75, *P* < 0.05). By contrast, the M1-stage patients did not change significantly (APC = −0.61, *P* > 0.05) (Figure 3(b)). These trends suggest that the incidence-based mortality has been significantly declining only in M0-stage gastric SRC patients after 2002.

### 3.5. Survival Trend for M0-Stage Gastric SRC

**Figure 4:**
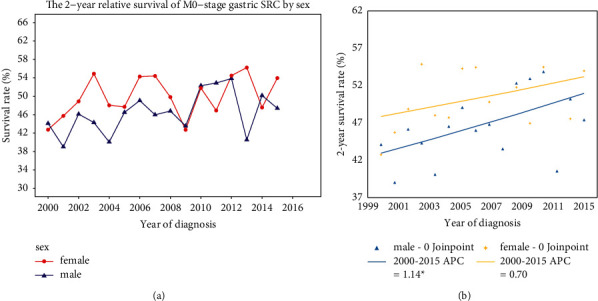
(a) The 2-year relative survival trends in M0-stage gastric SRC by sex. (b) Joinpoint analysis of the 2-year relative survival trend in M0-stage gastric SRC by sex.

We have also analyzed the 2-year relative survival trends in M0-stage gastric SRC. Specifically, the 2-year survival for M0-stage patients increased from 43.47% in 2000 to 50.42% in 2015. Further, we analyzed the differential trends by sex. The survival rate in males increased from 44.11% in 2000 to 47.39% in 2015. In parallel, the survival rate in females increased from 42.75% in 2000 to 53.95% in 2015 (Figure 4(a)). In joinpoint analysis by sex, the 2-year relative survival increased significantly in males (APC = 1.14, *P* < 0.05). However, the increase in this survival was not significant in females (APC = 0.7, *P* > 0.05) (Figure 4(b)). These trends indicate that 2-year relative survival has improved in M0-stage gastric SRC, especially for male patients.

### 3.6. Survival Trend for M1-Stage Gastric SRC

**Figure 5:**
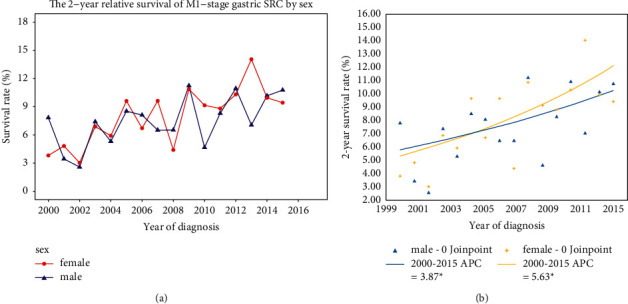
(a) The 2-year relative survival trends in M1-stage gastric SRC by sex. (b) Joinpoint analysis of the 2-year relative survival trend in M1-stage gastric SRC by sex.

With respect to the 2-year relative survival trend in M1-stage gastric SRC, this rate was also elevated during the study period. Despite the fluctuations over years, the overall trend of survival is upward for each sex. In 2000, only 7.83% of male patients and 3.81% of female patients relatively survived 2 years. In 2015, the survival rate increased to 10.77% in male and 9.43% in female (Figure 5(a)). In joinpoint analysis by sex, the 2-year relative survival significantly elevated in both male (APC = 3.87, *P* < 0.05) and female (APC = 5.63, *P* < 0.05) patients (Figure 5(b)). Before 2005, this survival rate was higher in males than in females, but this trend changed after 2005. The survival rate in males rose from 8.53% in 2005 to 10.77% in 2015. In parallel, 2-year relative survival rate in females increased from 9.61% in 2005 to 14.05% in 2013. These results indicate that the 2-year survival has significantly increased in the M1-stage gastric SRC patients over time.

## 4. Discussion

Gastric SRC is a distinct cancer subtype with a slowly increasing incidence [11]. In the context of changing epidemiology in America, we have analyzed the incidence-based mortality and survival trends in gastric SRC patients from 2000 to 2017. Our results demonstrate that the overall incidence-based mortality of gastric SRC has declined in America after 2002. When analyzing the incidence-based mortality of gastric SRC by sex and stage, we have found several distinct trends. First, the mortality rate was higher in males than females. Second, the mortality rate decreased significantly in males but insignificantly in females. Third, the M0-stage patients had a significantly reduced mortality rate, while the M1-stage patients had an insignificantly reduced mortality rate. The results of joinpoint analyses have confirmed these trends.

The overall incidence-based mortality from gastric SRC has declined after 2002. Much of this decline may be attributed to the recent advance in gastric cancer screening. Screening methods include imaging examination, electronic endoscopy, and magnetically controlled capsule endoscopy (MCE) [12]. Advanced endoscopy allows for detection of early-stage GC and curable premalignant lesions in the stomach [13]. The novel biomarkers identified in recent years may promote precision prevention and reduce mortality [12]. Moreover, the development of gene diagnosis can immediately affect the mortality rate of gastric cancer. The molecular characterization of human epidermal growth factor receptor 2 (HER2) expression in gastric cancer can guide anti-HER2 targeted therapy [14]. These methods can effectively reduce the overall mortality from GC.

The stratified analyses by sex produced similar results with a joinpoint at 2002, after which the mortality rates began declining. Although the incidence-based mortality is significantly higher in males than females, the rate decreased significantly in males rather than females. The faster abatement of mortality in males than in females may be owed to the higher attributable fraction in men. The results of the INT-0116 study support this speculation. It revealed that adjuvant radiochemotherapy significantly reduced the relapse of gastric cancer in patients after radical surgery [15]. However, the subgroup analysis indicated that 71.2% of the recruited patients were male. A recent study has analyzed the sex difference in the incidence of gastric cancer [16]. It demonstrated that the decline in gastric cancer incidence was accompanied by a widening sex difference over time. Men often had higher incidence of gastric cancer than women [16]. The higher incidence in males may engender higher mortality than in females. We also assessed the variability of incidence-based mortality across M-stage. Obviously, mortality is higher in M1-stage patients than in M0-stage patients in most years. The joinpoint analysis suggested that incidence-based mortality was declining only in M0-stage gastric SRC patients after 2002. The gastric endoscope techniques can detect and resect the tumor early, which may prevent the tumor's progression at an early stage [10]. However, such a technological advance is inappropriate for patients with distant metastasis. These findings emphasize the importance of gastric SRC screening with routine techniques to detect this cancer at an early stage.

With respect to the survival trend in gastric SRC patients over the past decades, we have found remarkable increases in the 2-year relative survival of gastric SRC patients. The improving survival may be attributed to the development of perioperative chemotherapy or adjuvant chemoradiotherapy in recent years [17]. A recent study indicated that advanced stage gastric SRC receiving TEFOX chemotherapy had an effective survival rate of 65%, and the median survival even reached 14 months [18]. Additionally, clinical cancer care has gradually shifted to multidisciplinary treatment in the past years, which has significantly improved the survival for these patients [19]. The significant survival improvement highlights the rapid changes in clinical practice for gastric cancer.

We have analyzed the different survival trends between males and females in M0-stage gastric SRC patients. The results indicated that 2-year relative survival was higher in females than in males during the study period but significantly improved only in male patients. A recent meta-analysis indicated that longer exposure to estrogen may alleviate the risk of gastric cancer [20]. Estrogen may increase the expression of trefoil factor proteins, which can inhibit oncogene expression and further protect the mucous epithelia [21]. This evidence may account for the higher relative survival in female patients. Nevertheless, women still face many obstacles, such as cancer screening, availability of healthcare, and social injustice [22]. These difficulties may lead to the insignificant survival improvement over years in female gastric SRC patients. In terms of the survival trend in M1-stage patients, our results indicated that 2-year relative survival has significantly improved both in male and female patients. Recent advances in targeted therapy, such as trastuzumab deruxtecan (T-DXd), have demonstrated significant survival benefits for HER-2 positive advanced gastric cancer [23]. More recently, immune checkpoint inhibitors have revolutionized the therapy for patients with advanced gastric cancer [24]. The phase II KEYNOTE-059 trial first evaluated the safety and efficacy of pembrolizumab in patients with metastatic gastric cancer [25]. This drug achieved higher objective response rates and longer response duration in PD-L1^+^ metastatic gastric cancer as a third-line treatment. Evidently, novel therapeutic approaches in the past decades have made a rich contribution to the prolonged survival in patients with gastric SRC.

Some limitations should be concerned. Given the time span of collected patients and different staging systems, some inevitable bias may exist in our analysis [26]. Moreover, some clinical variables, such as commodities, symptoms, and alcohol status, are not recorded in the SEER database. These factors may also influence the incidence-based mortality and survival trends of the patients. So, our results should be interpreted with caution. In spite of these limitations, our study still has several strengths. To our knowledge, this is the first study to comprehensively analyze the incidence-based mortality and survival trends in gastric SRC patients in America over the past decades. Our study was based on the high-quality SEER cancer registries with representative population-based data. The incidence-based mortality method has excluded the deaths from other cancers, allowing more specific analyses than death certificate-based estimation [19]. This method may more accurately reflect the real mortality trends of gastric SRC in the United States.

## 5. Conclusions

According to the analysis of the SEER database, we have found the declining incidence-based mortality of gastric SRC ever since 2002. The mortality decrease is significant in males and M0 stage patients. The survival of gastric SRC patients has improved significantly in the past decades. Such trends can be interpreted by the change in cancer screening and the advance in novel treatments.

## Figures and Tables

**Table 1:** Characteristics of patients with gastric SRC (*N* = 14916).

Stage	Sex, *n* (%)		
	Male	Female	Total, *n* (%)
M0-stage	4282 (54.89)	3784 (53.18)	8066 (54.08)
M1-stage	3519 (45.11)	3331 (46.82)	6850 (45.92)
Total, *n* (%)	7801 (52.3)	7115 (47.7)	14916 (100)

## Data Availability

The data are publicly available in the SEER database.

## References

[1] Nagtegaal I. D., Odze R. D., Klimstra D. (2020). The 2019 WHO classification of tumours of the digestive system. *Histopathology*.

[2] Assarzadegan N., Montgomery E. (2021). What is new in the 2019 world health organization (WHO) classification of tumors of the digestive system: review of selected updates on neuroendocrine neoplasms, appendiceal tumors, and molecular testing. *Archives of Pathology & Laboratory Medicine*.

[3] Zhang C., Liu R., Zhang W. H. (2021). Difference between signet ring cell gastric cancers and non-signet ring cell gastric cancers: a systematic review and meta-analysis. *Frontiers in Oncology*.

[4] Kiso M., Urabe Y., Ito M. (2020). Clinical and genomic characteristics of mucosal signet-ring cell carcinoma in Helicobacter pylori-uninfected stomach. *BMC Gastroenterology*.

[5] Kao Y.-C., Fang W.-L., Wang R.-F. (2019). Clinicopathological differences in signet ring cell adenocarcinoma between early and advanced gastric cancer. *Gastric Cancer*.

[6] Pernot S., Voron T., Perkins G., Lagorce-Pages C., Berger A., Taieb J. (2015). Signet-ring cell carcinoma of the stomach: impact on prognosis and specific therapeutic challenge. *World Journal of Gastroenterology*.

[7] Pokala S. K., Zhang C., Chen Z. (2018). Incidence, survival, and predictors of lymph node involvement in early-stage gastric signet ring cell carcinoma in the US. *Journal of Gastrointestinal Surgery*.

[8] Cardoso R., Guo F., Heisser T. (2021). Colorectal cancer incidence, mortality, and stage distribution in European countries in the colorectal cancer screening era: an international population-based study. *The Lancet Oncology*.

[9] Ayati A., Moghimi S., Toolabi M., Foroumadi A. (2021). Pyrimidine-based EGFR TK inhibitors in targeted cancer therapy. *European Journal of Medicinal Chemistry*.

[10] Li H., Zong Z., Zhou T. (2019). Trends of incidence and survival in patients with gastroenteropancreatic signet ring cell carcinoma: an analysis from the surveillance, epidemiology, and end results program. *Journal of Gastrointestinal Oncology*.

[11] Kanavati F., Ichihara S., Rambeau M., Iizuka O., Arihiro K., Tsuneki M. (2021). Deep learning models for gastric signet ring cell carcinoma classification in whole slide images. *Technology in Cancer Research and Treatment*.

[12] Fan X., Qin X., Qin X. (2021). Screening for gastric cancer in China: advances, challenges and visions. *Chinese Journal of Cancer Research*.

[13] Banks M., Graham D., Jansen M. (2019). British society of gastroenterology guidelines on the diagnosis and management of patients at risk of gastric adenocarcinoma. *Gut*.

[14] Wang F.-H., Shen L., Li J. (2019). The Chinese society of clinical oncology (CSCO): clinical guidelines for the diagnosis and treatment of gastric cancer. *Cancer Communications*.

[15] Smalley S. R., Benedetti J. K., Haller D. G. (2012). Updated analysis of SWOG-directed intergroup study 0116: a phase III trial of adjuvant radiochemotherapy versus observation after curative gastric cancer resection. *Journal of Clinical Oncology*.

[16] Lou L., Wang L., Zhang Y. (2020). Sex difference in incidence of gastric cancer: an international comparative study based on the global burden of disease study 2017. *BMJ Open*.

[17] Zhang S., Liu Y., Jiao Z. (2021). Development and validation of a prognostic nomogram for gastric signet ring cell carcinoma: a multicenter population-based study. *Frontiers in Oncology*.

[18] Pernot S., Dubreuil O., Aparicio T. (2018). Efficacy of a docetaxel-5FU-oxaliplatin regimen (TEFOX) in first-line treatment of advanced gastric signet ring cell carcinoma: an AGEO multicentre study. *British Journal of Cancer*.

[19] Burton A., Tataru D., Driver R. J. (2021). Primary liver cancer in the UK: incidence, incidence-based mortality, and survival by subtype, sex, and nation. *JHEP Reports: Innovation in Hepatology*.

[20] Camargo M. C., Goto Y., Zabaleta J., Morgan D. R., Correa P., Rabkin C. S. (2012). Sex hormones, hormonal interventions, and gastric cancer risk: a meta-analysis. *Cancer Epidemiology, Biomarkers & Prevention: A Publication of the American Association for Cancer Research, Cosponsored by the American Society of Preventive Oncology*.

[21] Chandanos E., Lagergren J. (2008). Oestrogen and the enigmatic male predominance of gastric cancer. *European Journal of Cancer*.

[22] Patel S., Pappoppula L., Guddati A. K., Annamaraju P. (2020). Analysis of race and gender disparities in incidence-based mortality in patients diagnosed with thyroid cancer from 2000 to 2016. *International Journal of General Medicine*.

[23] Kahraman S., Yalcin S. (2021). Recent advances in systemic treatments for HER-2 positive advanced gastric cancer. *OncoTargets and Therapy*.

[24] Ghidini M., Petrillo A., Botticelli A. (2021). How to best exploit immunotherapeutics in advanced gastric cancer: between biomarkers and novel cell-based approaches. *Journal of Clinical Medicine*.

[25] Fuchs C. S., Doi T., Jang R. W. (2018). Safety and efficacy of pembrolizumab monotherapy in patients with previously treated advanced gastric and gastroesophageal junction cancer: phase 2 clinical KEYNOTE-059 trial. *JAMA Oncology*.

[26] Wang P., Zhou H., Han G. (2021). Assessment of the value of adjuvant radiotherapy for treatment of gastric adenocarcinoma based on pattern of post-surgical progression. *World Journal of Surgical Oncology*.

